# Effect of Radiation Dose-Rate on Hematopoietic Cell Engraftment in Adult Zebrafish

**DOI:** 10.1371/journal.pone.0073745

**Published:** 2013-09-18

**Authors:** Tiffany J. Glass, Susanta K. Hui, Bruce R. Blazar, Troy C. Lund

**Affiliations:** 1 Department of Molecular, Cellular, Developmental Biology and Genetics, University of Minnesota Medical School, Minneapolis, Minnesota; 2 Department of Therapeutic Radiology-Radiation Oncology, University of Minnesota Medical School, Minneapolis, Minnesota; 3 Department of Pediatrics, Division of Hematology, Oncology and Transplantation, University of Minnesota Medical School, Minneapolis, Minnesota; UMDNJ-Robert wood Johnson Medical School, United States of America

## Abstract

Although exceptionally high radiation dose-rates are currently attaining clinical feasibility, there have been relatively few studies reporting the biological consequences of these dose-rates in hematopoietic cell transplant (HCT). In zebrafish models of HCT, preconditioning before transplant is typically achieved through radiation alone. We report the comparison of outcomes in adult zebrafish irradiated with 20Gy at either 25 or 800 cGy/min in the context of experimental HCT. In non-transplanted irradiated fish we observed no substantial differences between dose-rate groups as assessed by fish mortality, cell death in the kidney, endogenous hematopoietic reconstitution, or gene expression levels of *p53* and *ddb2* (damage-specific DNA binding protein 2) in the kidney. However, following HCT, recipients conditioned with the higher dose rate showed significantly improved donor-derived engraftment at 9 days post transplant (p≤0.0001), and improved engraftment persisted at 31 days post transplant. Analysis for *sdf-1a* expression, as well as transplant of hematopoietic cells from *cxcr4b −/−* zebrafish, (*odysseus*), cumulatively suggest that the *sdf-1a/cxcr4b* axis is not required of donor-derived cells for the observed dose-rate effect on engraftment. Overall, the adult zebrafish model of HCT indicates that exceptionally high radiation dose-rates can impact HCT outcome, and offers a new system for radiobiological and mechanistic interrogation of this phenomenon. Key words: Radiation dose rate, Total Marrow Irradiation (TMI), Total body irradiation (TBI), SDF-1, Zebrafish, hematopoietic cell transplant.

## Introduction

Radiation preconditioning is often used to prepare patients for hematopoietic cell transplant (HCT), and is also a common preconditioning method in animal models used to study the mechanisms of hematopoietic cell homing and engraftment. Radiation dose-rates for clinical preconditioning total body irradiation (TBI) commonly range from 5–30 cGy/min [1]. However, clinical feasibility and efficacy have recently been demonstrated for alternative radiation delivery methods capable of markedly higher dose rates, up to 800 cGy/min [2], [3]. Although these higher radiation dose-rates have been shown to be compatible with hematopoietic engraftment in humans, it is unknown whether they can have unique impacts on the marrow microenvironment or kinetics of hematopoietic engraftment.

Historically, studies comparing effects of radiation dose-rate for TBI at ranges substantially lower than 800 cGy/min found strong correlations between higher dose-rates and non-hematopoietic toxicity, with dose-rate effects on hematopoietic damage observed to be less striking than the adverse dose-rate effects on organs such as lung and gastrointestinal tract [4], [5]. However, despite reductions in off-target toxicities associated with lowered dose-rates, delivery of a desired dose at a low rate during a single treatment session could present logistical drawbacks due to prohibitively long delivery times [6]. Early recognition of this helped to promote fractionated delivery, with goals to minimize organ toxicity, while leaving hematopoietic ablation effects essentially intact [6]. Today, new advances in conformal radiation now allow exceptionally high dose-rates targeted to the bone marrow alone, while sparing non-target organs [7]. This makes it possible to consider the therapeutic outcomes of high dose-rates that were traditionally considered inadvisable, and prompts renewed interest in refining our understanding of radiation dose-rate effects as they relate specifically to HCT and engraftment.

Anticipating the consequences of increasing radiation dose-rates in a biological process as complex as HCT is not necessarily straightforward. Inverse correlations have been reported between dose-rate and the dose required for engraftment after HCT. In murine models comparing dose-rate ranges at and below 50 cGy/min, this effect has been attributed to the capacity of the higher dose-rates to cause greater lethality to recipient proliferating T-lymphocyte precursors [8], [9]. However, slightly improved engraftment caused by higher dose-rates has been reported to occur in syngenic transplants as well [8], raising the possibility of unknown alternative mechanisms enforcing this dose-rate dependent outcome. Additionally, although it seems reasonable to expect greater recipient cell lethality after higher dose-rates, there are some experimental contexts in which moderately higher dose-rates actually cause significantly less irradiated cell lethality than very low dose-rates, arguably due to more efficient DNA damage detection and repair responses elicited by higher rates of damage [10].

Transplant of adult zebrafish can be used to experimentally model HCT. The primary hematopoietic organ in the zebrafish is the kidney, and the use of radiation to precondition adult zebrafish for HCT of donor hematopoietic cells obtained from the kidney is well established [11]–[13]. Zebrafish show evidence for conservation of mammalian DNA damage recognition and repair mechanisms, including those dependent on p53, and can additionally be used to screen for chemical modifiers of radiation sensitivity [14]–[17]. However, little is known about the impact of different radiation dose-rates on this vertebrate model. In this study, we compared adult zebrafish preconditioned with 20 Gy delivered with dose-rates of either 25 cGy/min, or 800 cGy/min, to determine the impacts, if any, of the dose-rates on this experimental system. Although indicators of radiation-induced toxicity and myelosupression in the kidney were observed to be similar in both the 800 cGy/min and 25 cGy/min dose-rate conditions, we found that recipients irradiated at the higher dose rate showed significantly greater levels of donor-derived cells beginning at 9 days post transplant (dpt), compared to recipients irradiated at the lower dose-rate.

## Materials and Methods

### Zebrafish care

Wild type (WT), transgenic Tg(*bactin2:EGFP*) [18], Tg(*h2afv:EGFP*) [19], Tg(*sdf-1a:DsRed*) [13], and *odysseus* mutant [20] zebrafish were housed in the University of Minnesota Zebrafish Core Facility according to previously described standards of care [21]. This study used only adult zebrafish, ranging in age from two months to approximately two years. The methods used in this work were approved by the *Institutional Animal Care and Use Committee* (*IACUC*), University of Minnesota.

### Radiation Delivery to Zebrafish

20 Gy radiation is a sublethal dose for adult zebrafish [12]. All experiments reported here used a total dose of 20 Gy, and dose-rates of either 25 cGy/min, or 800 cGy/min, to irradiate adult zebrafish in fish water within a large petri dish. For all radiation sessions, the petri dish was placed on 5 cm thick solid water material to prevent scattering. A 1.5 cm thick tissue-equivalent bolus for dose build up was placed on top of the petri dish. Dose was delivered with a Varian 2300CD linear accelerator (Varian, CA). The inverse square relation were used to determine the correct surface to source distance (SSD) for the desired dose rates based on the dose rate delivered at 100 cm SSD.

For radiation delivery at the 25 cGy/min dose rate, we used a SSD of 200 cm between the source and the surface of the bolus. The linear accelerator was programmed to deliver 100 cGy/min at 100 cm SSD (by setting 100 monitor unit or MU/minute). This achieved a dose rate of 25 cGy/min. For radiation delivery at dose rate of 800 cGy/min, SSD was kept at 87 cm between the source and the bolus, and the linear accelerator was programmed to deliver 600 cGy/min at 100 cm SSD. This achieved a dose-rate of 800 cGy/min.

### Dose verification and dose-rate verification

Dose-rates were verified with routinely used in-house ionization chamber [PTW, TN30013] and an electrometer [Keithley 602]. The dose homogeneity across the petri dish, measured using the extended dose rate (EDR) film, was observed to be within 5%. The ionization chamber was placed within solid water material, positioned 5 mm below the petri dish.

For the high dose-rate (800 cGy/min), the dose-rate was measured to be 0.217×10^−8^ Ampere (varied between 0.216 to 0.218), and the total beam on time was 2.5 minutes. For the low dose-rate (25 cGy/min), the dose-rate was measured to be 0.68×10^−10^ Ampere (varied between 0.660 to 0.704), and the total beam on time was 82.1 minutes. The dose-rate ratio measured using electrometer reading between high dose-rate and low dose-rate delivery was 0.217×10^−8^/0.68×10^−10^ = 31.9. This is equivalent to the expected dose-rate ratio of 32 (800/25), i.e., the time required to deliver the low dose-rate irradiation was 32 times greater than the time required to deliver the high dose-rate irradiation.

### Hematopoietic cell transplantation (HCT)

Two days following irradiation, HCT was performed by previously described methods [12]. Briefly, donor fish were euthanized in tricaine, beheaded, flushed with Phosphate Buffered Saline (PBS), and kidneys were removed into sterile 1X PBS. Kidneys were triturated, and filtered through a 40 µM filter to isolate whole kidney marrow cells (WKM). WKM cells were pelleted, resuspended in PBS, counted, and transplanted into anesthetized recipient fish through cardiac injection. Each recipient fish received a transplant cell dose of 400,000 WKM cells. Donor cells were harvested from either Tg(*bactin2:EGFP*) (in which the *bactin2* promoter drives EGFP expression in many, but not all, cells) [18] [12] [22], or Tg(*h2afv:EGFP*) (in which the *h2afv* promoter drives EGFP expression in essentially all hematopoietic cells) [19]. Within each transplant experiment comparing dose-rate groups, both recipient dose-rate groups received transplants from the same pool of donor cells. Recipient fish were either WT, or Tg(*sdf-1a:DsRed*); in which sites of DsRed expression include subsets of renal tubules [13].

### Hematopoietic cell analysis

Viability of WKM cells was determined by staining with Propidium Iodide (BD Phamingen) and analyzed by flow cytometry, using a BD FacsCanto. Hematopoietic cells (myelomonocytes, lymphocytes, precursors) were identified with the FSC/SSC characteristics as previously described [12], and donor-derived hematopoietic cells were identified through GFP expression. 100,000 to 250,000 events were collected per sample. Flow cytometry experiments analyzed each individual fish separately; specimens were not pooled for data acquisition.

### TUNEL Staining

TUNEL staining was performed with an In Situ Cell Death Detection kit, POD (Roche). One day after radiation, fish kidneys were harvested and fixed overnight in 10% Neutral Buffered Formalin (Sigma), then rinsed in PBS, and soaked in 30% sucrose, 10% OCT overnight. Specimens were transferred to OCT, and slides with 8 µM sections were processed for cell death detection according to manufacturer guidelines. Tissue sections from three fish per experimental group were analyzed.

### qRT-PCR of zebrafish tissues

Isolation of WKM and renal tubules from zebrafish kidneys was performed as described previously [13]. Briefly, zebrafish kidneys are triturated in PBS and pipetted onto a 40 µM nylon cell strainer. Hematopoietic cells (WKM) pass through the strainer and are collected. The strainer is then inverted, and PBS is pipetted through it. This releases renal tubules, which were too large to pass through the strainer. Collected cell samples are then centrifuged at 2000 g for 5 minutes to obtain cell pellets. RNA isolation and DNA removal were achieved with the RNeasy Mini Kit (Qiagen). cDNA was synthesized using SuperScript III 1^st^ strand synthesis kit (Invitrogen). qRT-PCR was performed using Taqman Primers (listed in supplemental data) and reagents (Applied Biosystems). qRT-PCR reactions were performed in technical triplicates on a StepOnePlus qRT-PCR system (Applied Biosystems). Each biological sample analyzed by qRT-PCR was composed of tissues pooled from 4 adult fish. Relative quantitation (RQ) values were determined through the δ-δ-C_t_ method. *Bactin1* was set as the control gene in all experiments.

### Microphotography and Image Analysis

Single-channel photographs were acquired using Leica LAS software, DFC340FX camera, Leica MZ6 stereomicroscope, and Leica GFP filter cube. Specimens too large to fit within a single field of view were photographed across the xy plane as needed, and photomontages were manually assembled in Adobe Photoshop CS4. Where indicated, quantitative assessment of Green Fluorescent Protein (GFP) signal was accomplished using one photo composite of each fish, cropped to isolate the body only, and analyzed for the mean gray value in ImageJ. Dual-channel photographs were obtained using a DMI6000B Leica microscope, Retiga2000R camera, and QCapture Basic acquisition software. Adobe Photoshop CS4 was used to pseudocolor and manually assemble photo composites.

### Statistical Analysis

GraphPad Prism was used to perform statistical analysis. qRT-PCR data was analyzed by repeated measures ANOVA and Tukey's post comparison test. Unpaired t-tests were used to analyze flow cytometry assessments of hematopoietic cells.

## Results

### Effects of Dose-Rates on Non-Transplanted Zebrafish

**Figure 1:**
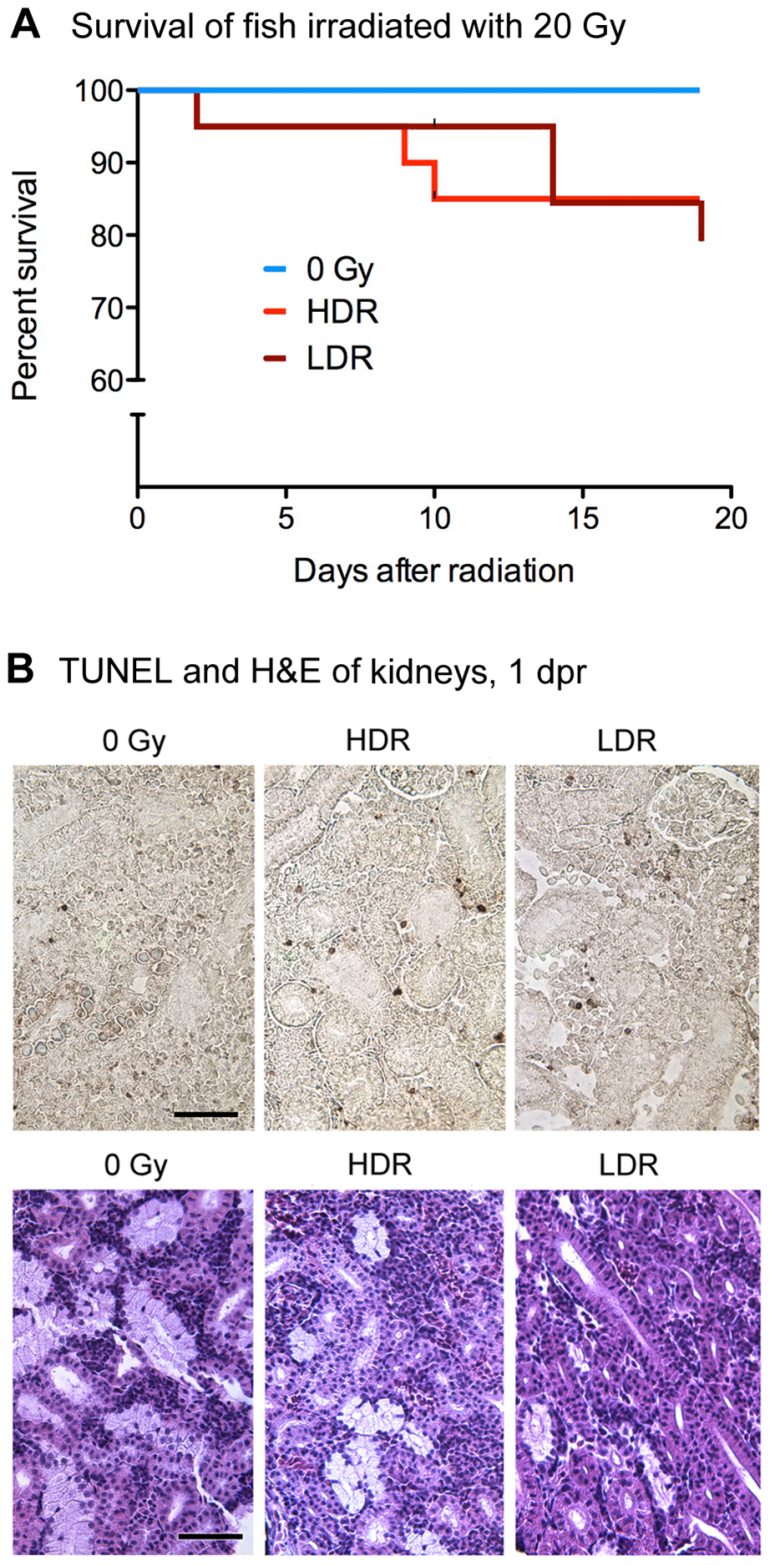
Consequences of 20Gy at 25 cGy/min vs 800cGy/min in non-transplanted fish. A, Kaplan-Meier survival curves of adult zebrafish irradiated with 20Gy at either 25cGy/min (LDR) or 800cGy/min (HDR). Pooled data from two independent trials, each trial composed of ten fish per group. p = .356 (Log Rank Test). B, TUNEL staining (above), and H&E staining (below) of adult zebrafish kidneys harvested one day after 20Gy radiation delivered at either 25cGy/min or 800cGy/min.

**Figure 2:**
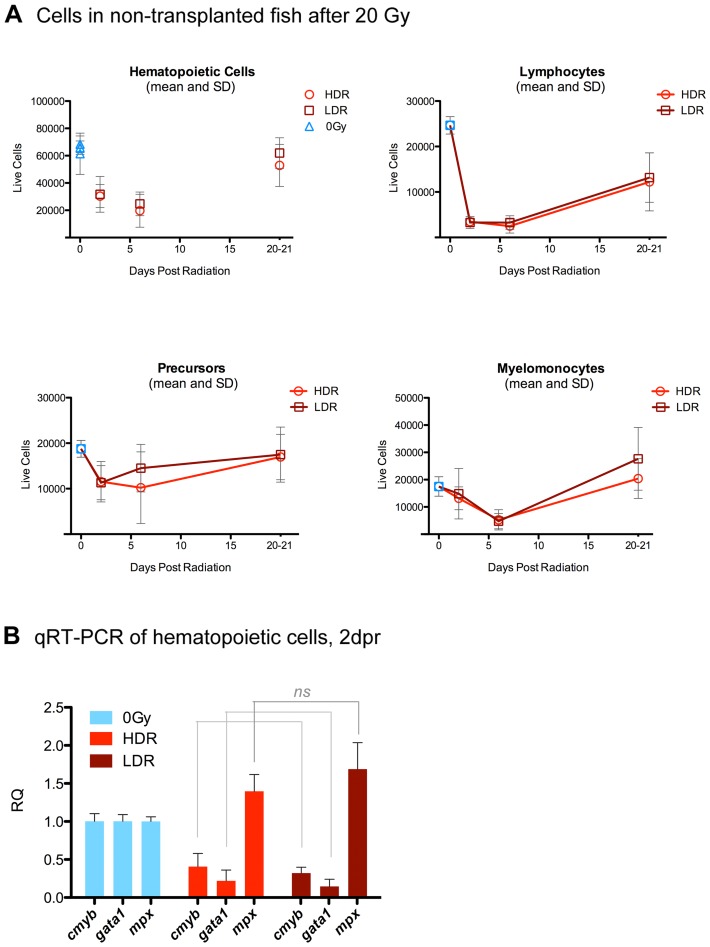
Myelosuppression after 20Gy at 25 cGy/min vs 800cGy/min in non-transplanted fish. A, Flow cytometry analysis of hematopoietic cell recovery in the kidney on days 2, 6, and 20–21 following radiation at either 25cGy/min (LDR) or 800cGy/min (HDR), showing initial myelosuppression followed by hematopoietic recovery. Plot shows the means and SDs of pooled data from two independent biological trials, for a total of at least 7–10 fish per data point. 100,000 events were collected per sample. No significant differences were noted between dose-rate groups. B, qRT-PCR of hematopoietic cells 2 days post radiation. Data shows pooled results from two independent biological trials; each trial composed of 4 fish per group. No statistically significant differences between dose-rate groups were noted. RQ: Relative Quantitation.

In order to compare the relative impacts of 25 cGy/min and 800 cGy/min dose-rates on fish irradiated with 20 Gy, we performed survival, cell death, and myelosuppression studies on irradiated, non-transplanted adult WT zebrafish. Two independent trials, each composed of 10 fish per group, revealed no significant differences between dose-rate groups in survival over 19 days (Figure 1A). TUNEL staining and H&E in kidney sections one day after radiation suggested similar levels of apoptosis and hematopoietic cell loss, respectively, in both dose-rate groups (Figure 1B, and Figure S1). Flow cytometry analysis of WKM isolated from WT fish at multiple time points following radiation revealed hematopoietic cell loss and subsequent recovery, as has been previously reported to occur following sublethal irradiation of fish [12]. However, there were no significant differences between dose-rate groups in the relative numbers of viable hematopoietic cells present in the kidney during this endogenous reconstitution process. Although some fish irradiated at the lower dose-rate were found to have slightly higher means of some cell types at days 6 and 21, these differences were not significant (Figure 2A). Similarly, qRT-PCR analysis of hematopoietic cells isolated 2 days after radiation from non-transplanted, irradiated fish revealed no significant differences between dose-rate groups in expression levels of *c-myb, gata1*, or *mpx* (Figure 2B). Transcription factor *cmyb* has been previously reported to be expressed in HSC [23], [24], *gata1* expression is a hallmark of erythroid lineage cells [25], [26], and *mpx* is expressed by myeloid lineage cells [26], [27]. These results are compatible with similar levels of myelosuppression between dose-rate groups during the time-point at which experimental transplant typically occurs. Cumulatively, these results provide no evidence for differences in myelosuppression between dose-rate groups.

**Figure 3:**
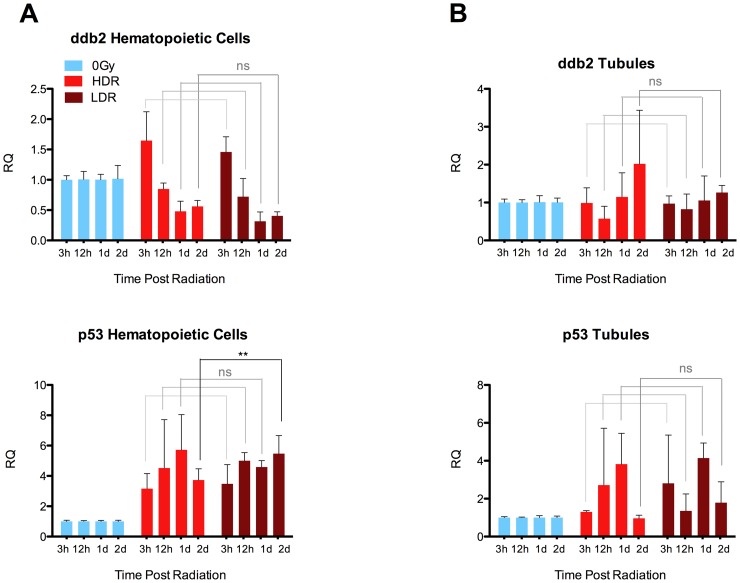
qRT-PCR of hematopoietic and non-hematopoietic kidney after radiation. 3/min (HDR) or a lower dose rate of 25 cGy/min (LDR), WT zebrafish kidneys were pooled and filtered to obtain samples enriched for either hematopoietic whole kidney marrow (WKM) (A), or renal tubules (B). 0 Gy control samples were harvested concurrently at each time point and set as biological references. Each biological trial was composed of kidneys from four fish per time point, and data summarizes pooled results from 2–3 independent biological trials per time point, per condition. Although LDR hematopoietic cells showed significantly higher *p53* expression 2 dpr than HDR hematopoietic cells (.001<P<.01), this was less than a 2-fold difference. No other significant differences were found between dose rate groups. RQ: Relative Quantitation, *bactin1* controls.

We next assessed damage-response gene expression changes at several time points ranging from 3 hours to 2 days after radiation delivered at each of the dose rates. After radiation, samples of pooled zebrafish kidneys were processed to separate the tissues into a fraction enriched for hematopoietic cells from the kidney, and a fraction enriched for the non-hematopoietic (primarily renal tubule) cells. These fractions were then assessed separately for *ddb2* and *p53* gene expression changes. *Ddb2* is a gene involved in DNA repair which has been previously reported to undergo significant expression level changes in human radiation therapy patients, and has consequently been proposed to show potential as a biomarker of radiation response [28], [29]. Although zebrafish hematopoietic cell samples showed radiation-induced changes in expression levels following radiation, no significant differences were found between the two dose-rate groups before and up to one day after radiation. While *p53* was found to be significantly up-regulated in LDR hematopoietic cells 2 days after radiation, the amount of up-regulation was lower than 2-fold and therefore potentially biologically insignificant (Fig. 3A). Similarly, sample fractions enriched for renal tubules showed no significant differences in gene expression changes between the two dose-rate groups (Fig. 3B). Although no remarkable differences between dose-rates were observed in p53 expression levels, upregulation of p53 occurred at several time points after radiation, and this was a more consistent finding in hematopoietic cells than in tubules. Cumulatively, these results suggest that 20 Gy delivered at 800 cGy/min causes damage profiles that are in some aspects similar to the damage profiles caused by 20 Gy delivered at 25 cGy/min.

### Effects of Dose Rates on Hematopoietic Engraftment

**Figure 4:**
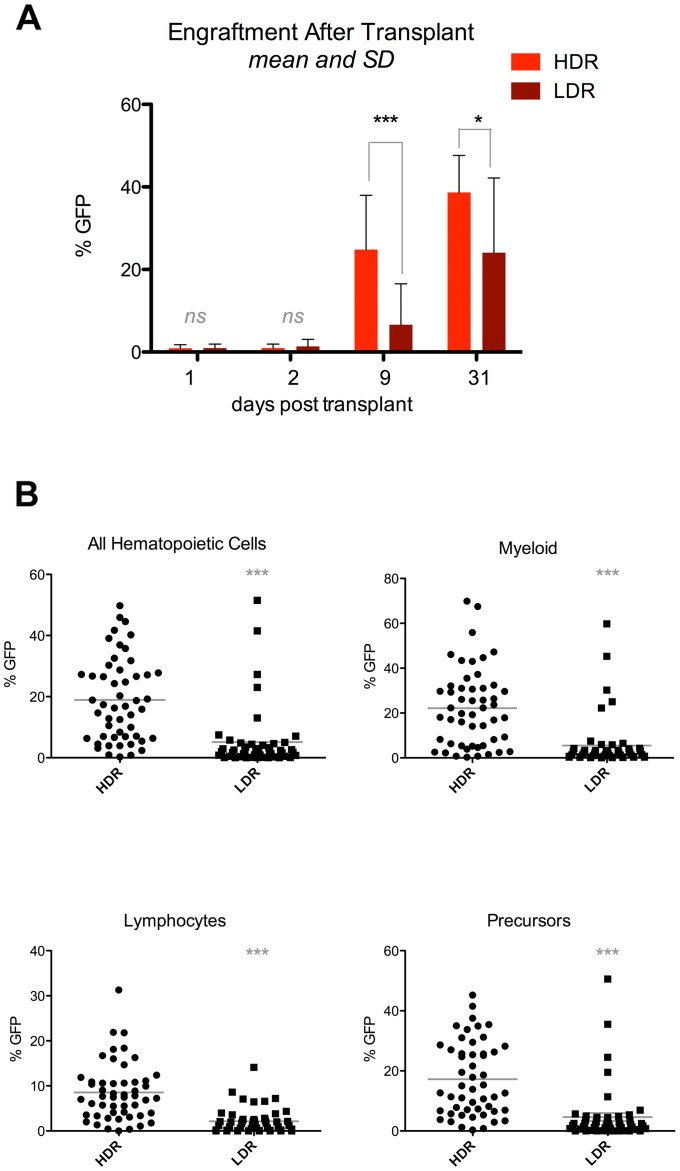
Hematopoietic cell transplant after radiation. A, WT zebrafish were irradiated with 20Gy at either 800/min (HDR) or 25 cGy/min (LDR), and transplanted with *bactin2:EGFP* hematopoietic cells. At 1, 2, 9, and 31 days after transplant, kidneys were harvested from recipients and isolated hematopoietic cells were analyzed by flow cytometry for percentages of GFP-positive cells. Data shows results from at least two independent biological experiments per timepoint, for totals of: 14 fish per group (1 dpt), 12–13 fish per group (2 dpt), 25–26 fish per group (9dpt), and 10–13 fish per group (day 31). 9 dpt p<0.0001, 31 dpt p = 0.0301. Bars show mean and SD. B, Flow cytometry analysis of hematopoietic cells isolated from transplanted fish 9 days after transplant. Each data point indicates results from one fish. Data is pooled from six biologically independent experiments; three with *bactin2:EGFP* donor cells and three with *h2afv:EGFP* donor cells, for cumulative totals of 51 and 48 fish in the HDR and LDR groups, respectively. p<0.0001. Bars show the mean.

In order to assess the relative impacts of these radiation dose-rates on HCT in this zebrafish model, we irradiated recipients with 20 Gy at either 25 cGy/min or 800 cGy/min, transplanted each group with donor WKM from transgenic donors that constitutively expressed GFP, and used flow cytometry analysis of recipient kidneys at several time points after transplant to compare donor cell homing and engraftment between the two groups. No differences in homing were detected between dose-rate groups, as assessed at 1 and 2 days post transplant (Figure 4A). However, at nine days post transplant, recipients in the 800 cGy/min groups consistently contained significantly greater percentages of donor-derived cells compared to fish in the 25 cGy/min group (Figure 4A–B). Similarly, at 31 days post transplant, recipients in the higher dose-rate group showed significantly higher engraftment than recipients in the lower dose-rate group. (Figure 4A).

### Anatomical Characteristics of Engraftment 9 Days Post Transplant

**Figure 5:**
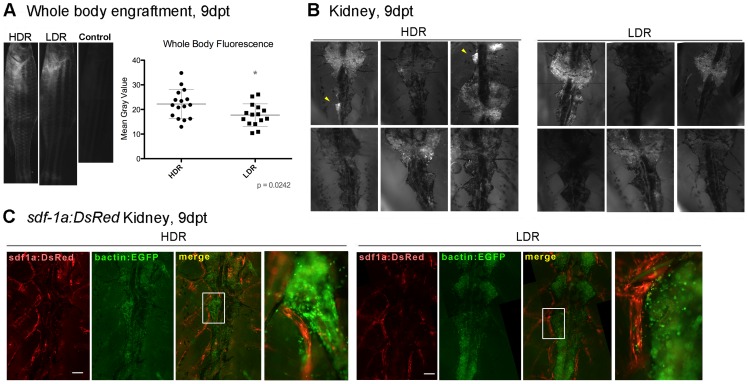
Anatomical characteristics of hematopoietic reconstitution 9dpt. A) Fish were irradiated with 20 Gy at either a high dose rate of 800 cGy/min (HDR) or lower dose rate of 25 cGy/min (LDR), and transplanted with *Bactin2:EGFP* WKM cells. 9 days after transplant (dpt), live fish were photographed to record whole-body GFP signal. Mean gray values were obtained from manual photo composites of each fish. Representative photographs show the approximate mean gray value of each group. Data shows results pooled from two independent biological experiments, for a composite total of 15–16 fish per group. B) Fresh kidneys were photographed to detect GFP signal 9 dpt, six fish per group. Sites with exceptionally high concentrations of donor-derived cells are indicated with yellow arrowheads. C) Tg(*sdf-1a:DsRed*) recipients were transplanted with *bactin2:EGFP* WKM cells. 9 dpt, kidneys from each group were photographed. Manual photo composites document *sdf-1a:DsRed* and *bactin2:EGFP* expression. Region of enlargement was chosen based on anatomical similarity to indicated regions in panel B. No HDR-specific expression patterns of *sdf-1a* were noted. Kidneys of three recipients per group were photographed and analyzed. Scale, 200 µM.

Because dose-rate was found to impact hematopoietic reconstitution as early as 9 dpt, fluorescence microscopy was used to assess localization of donor-derived cells at that time point. Whole body fluorescence detection of the living fish showed donor-derived cells distributed throughout the body in both dose-rate groups at 9 dpt. Recipients irradiated at 800 cGy/min showed significantly increased levels of donor-derived fluorescence signal (Figure 5A). 9 days after WT fish were transplanted with GFP-labeled cells, *ex vivo* preparations of their kidneys periodically reveal highly localized concentrations of donor-derived cells (Figure 5B). Because previous studies have suggested renal tubule cells may help form the hematopoietic stem and progenitor cell niche in this organism [13], [30], and might therefore be expected to support localized hematopoietic proliferation, we next assessed kidney preparations of Tg(*sdf-1a:DsRed*) transplant recipients. Tg(*sdf-1a:DsRed*) fish allow fluorescence detection of subsets of renal tubules that express high levels of *sdf-1a*. However, despite the presence of donor-derived cells adjacent to *sdf-1a:DsRed* structures in both groups, anatomical expression patterns of *sdf-1a* expression in the kidney did not appear to uniquely correlate with dose-rate (Fig. 5C).

### The *sdf-1a/cxcr4b* Axis is Not Required for Improved Engraftment 9 dpt in Recipients Conditioned at 800 cGy/min

**Figure 6:**
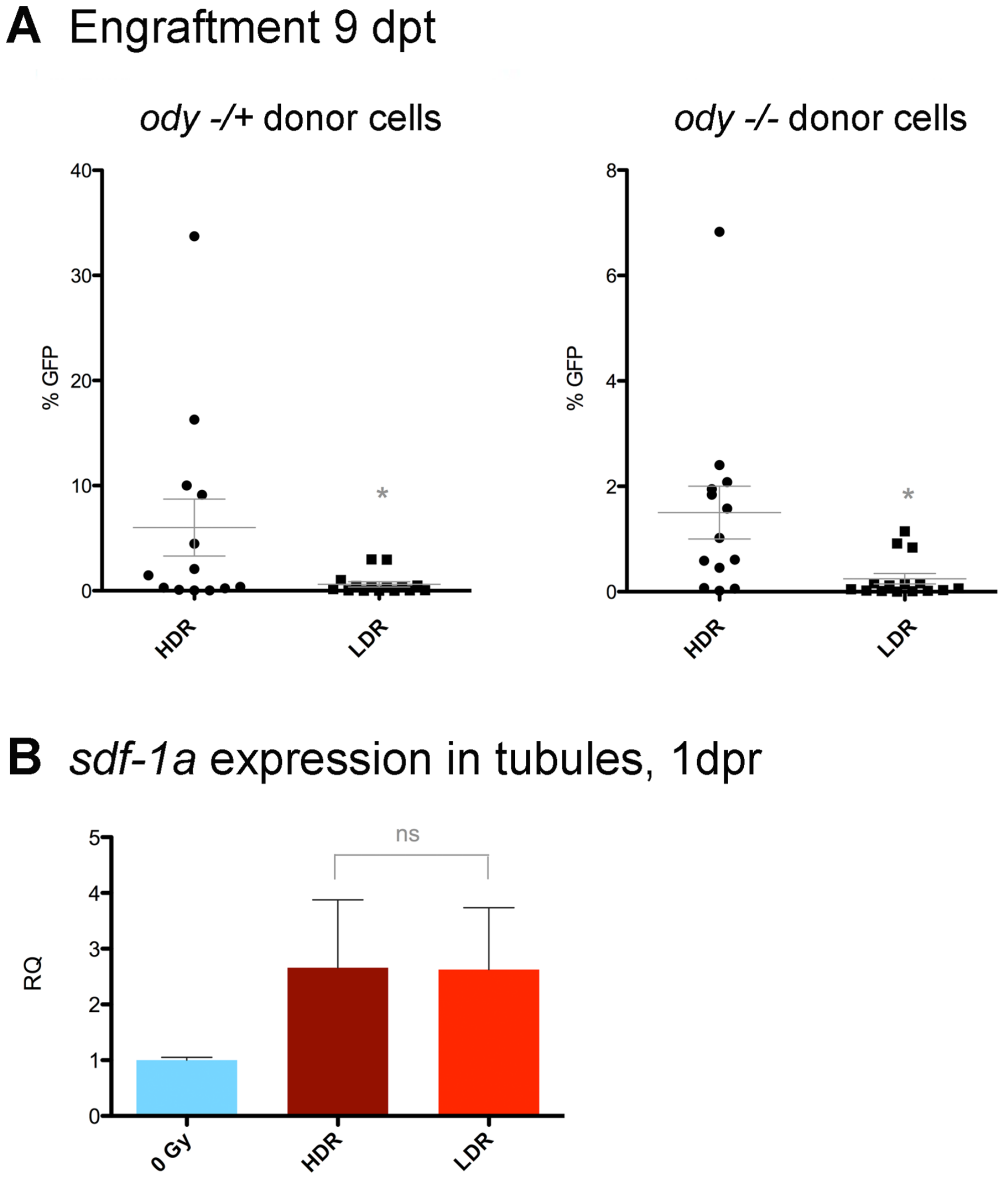
Dose-Rate effect does not require cxcr4b in transplanted cells. A) WT zebrafish were irradiated with 20 Gy at either dose rate and transplanted with WKM from Tg(*bactin*:EGFP) WKM that was either heterozygous or homozygous for the *odysseus* (*cxcr4b*) mutation. 9 dpt, kidneys were harvested and analyzed by flow cytometry. Data shows pooled results from two biologically independent trials, n = 13–15 fish per group. Heterozygous: p = 0.0420, Homozygous mutant: p = 0.0141, (unpaired t-test). B) 1 day after radiation, samples enriched for renal tubules were collected from WT zebrafish and analyzed by qRT-PCR for *sdf-1a* expression. No significant differences were seen between dose-rate groups. Pooled results from two biological experiments, each with four fish per group, are shown. RQ: Relative Quantitation.

In light of the fact that previous studies have reported radiation-induced changes in *sdf-1* expression levels [13], and reports have also suggested roles for SDF-1 in hematopoietic cell survival and proliferation [31], we next sought to assess the functional relevance of this chemokine to the improved engraftment 9 dpt in high dose-rate conditioned fish. To this end, WT zebrafish were preconditioned as before and transplanted with Tg(*bactin2:EGFP*) cells that were also either heterozygous or homozygous for the loss of function in the Cxcr4b receptor for Sdf-1a (*odysseus* mutation). Representative facs plots of heterozygous and homozygous odysseus donor WKM cells are shown in Figure S2. 9 days after transplant, recipient kidneys were harvested and assessed by flow cytometry. Both heterozygous and homozygous *odysseus* hematopoietic transplants showed greater percentages of donor-derived cells 9 dpt in the high dose-rate groups (Figure 6A). Additionally, qRT-PCR assays of irradiated, non-transplanted fish detected no significant differences in expression levels of *sdf-1a* between irradiated WT fish in the two dose-rate groups (Figure 6B). As donor cells insensitive to the *sdf-1a/cxcr4b* axis were capable of showing the dose-rate effect nine days after transplant, and comparison of the two dose-rates in non-transplanted fish revealed no significant differences in *sdf-1*a levels induced by the preconditioning rates, we conclude that the *sdf-1a/cxcr4b* axis is likely not required for the observed impact of radiation dose-rate on engraftment.

## Discussion

This study compared outcomes in adult zebrafish conditioned with a sublethal radiation dose delivered at either 25 cGy/min or 800 cGy/min. While striking differences between the dose-rate groups in parameters of cell death, myelosuppression, selected gene expression indicators of damage, and hematopoietic cell homing were not detected at early time points after radiation, recipient fish did show a significant dose-rate dependent effect in engraftment 9 and 31 days after HCT. At the earlier time point of 9 dpt, the higher dose-rate group showed better engraftment than the lower dose-rate group, indicating striking enhancement of early engraftment enabled by the higher dose-rate preconditioning. Our findings of biologically comparable outcomes in the two dose-rate groups at time points 1–2 days after radiation are consistent with the findings of a previous study of radiation dose-rate and HCT in mice, which found dose-rates ranging from 10 cGy/min to 585 cGy/min produced no substantial differences between groups in homing of lineage-depleted cells 2 dpt, but did show a small increase in donor-derived cells in bone marrow 5 dpt in the highest dose-rate group [32]. This study as well as our own suggest that salient effects specific to radiation dose-rate effects in transplanted organisms may not be readily detectable until several days after transplant. The mechanisms responsible for these effects are somewhat unclear.

In HCT, conditioning radiation initially causes cell death and myelosuppression, also elicits responses from the recipient tissues that can alter donor-derived cell migration and engraftment [34] [35] [36], and thirdly can cause both acute and long-term off-target toxicities and pathologies [37], [38]. It is conceivable that radiation dose-rate can modulate all three of these processes. However, our investigations thus far have not revealed significant differences in myelosuppression or early toxicity between the radiation dose-rate groups in the adult zebrafish model. In the future, refined assessment of dose-rate impacts on more specific hematopoietic stem and progenitor cell populations in the zebrafish may provide a better understanding of the mechanisms for the dose-rate effect on engraftment in this system. The likelihood that higher dose-rates can more effectively suppress important functional subsets of recipient immune cells merits continued recognition in light of previous work linking higher radiation dose-rate to more significantly improved engraftment in allogeneic, rather than syngenic, transplants [8]. Our findings of significantly fewer donor-derived cells in LDR groups at 9 dpt, but not at 1–2 dpt, are also compatible with a transplant rejection process occurring in LDR, but not HDR groups.

Apart from toxicity and myelosuppression, it has yet to be determined whether very high dose-rates can induce changes in the microenvironment of the hematopoietic tissues by modulating factors that direct donor cell migration, behavior, and proliferation, thereby influencing rate of engraftment. Sdf-1 has been previously shown to elicit homing in this model system [13]. However, we find that upregulation of *sdf-1* is not significantly different between the high and low dose-rate groups, nor do we observe differences in homing between the dose-rate groups. Combined with the finding that a dose-rate effect is essentially preserved in transplants of *odysseus* cells insensitive to Sdf-1a, it is reasonable to conclude that donor cell responses to the *sdf-1a/cxcr4* axis are probably not required for an effect of relatively improved engraftment 9 dpt caused by the higher dose-rate. However, although the *sdf-1a/cxcr4* axis may not be strictly required for this dose-rate effect, the possibility certainly remains that it may be quite relevant to the modulation of this effect. Sdf-1 is an important factor for several processes necessary for HCT success, including cell migration, hematopoietic differentiation, and reconstitution [13], [39], [40]. Therefore, despite our finding at 9 dpt that the dose-rate effect is essentially preserved when donor cells have loss of Cxcr4b function, it is still very likely that the mutant cells have an ameliorated capacity for fully normal, robust hematopoietic reconstitution after transplant.

Zebrafish do show some significant differences from mammals in response to radiation, due at least in part to the radioprotective consequences of being cold-blooded, as has been discussed previously [12]. However, as previous studies report similarities between zebrafish and humans in several aspects of hematopoietic biology that are highly relevant to HCT outcomes [13], [41] it is reasonable to expect that the zebrafish transplant model will continue to be informative to future dose-rate investigations.

## Supporting Information

Figure S1 **Quantification of TUNEL staining in Kidney sections.** Photographs of head, saddle, and tail kidney regions were taken using a 40X objective. The numbers of TUNEL-positive cells were manually counted in each photograph. 3 fish and ten photographs were analyzed per group. Bars show mean and SEM. No significant difference was noted (unpaired t-test).
(TIF)

Figure S2 **Hematopoietic Profiles of WT, ody −/+, and ody −/− Fish.** Facs plots of hematopoietic cells pooled from kidneys of several fish of each genotype. Although heterozygous odysseus fish and WT fish both show lymphocyte, precursor, and myelomonocyte populations, myelomonocytes are conspicuously reduced in kidneys of homozygous odysseus mutants.
(TIF)

Supporting Text S1 **Primers.**
(DOCX)
