# Supplementary figures and images for: Effect of Radiation Dose-Rate on Hematopoietic Cell Engraftment in Adult Zebrafish
Source: PLoS One. 2013 Sep 18;8(9):e73745. doi: 10.1371/journal.pone.0073745 (PMC3776794; doi:10.1371/journal.pone.0073745)

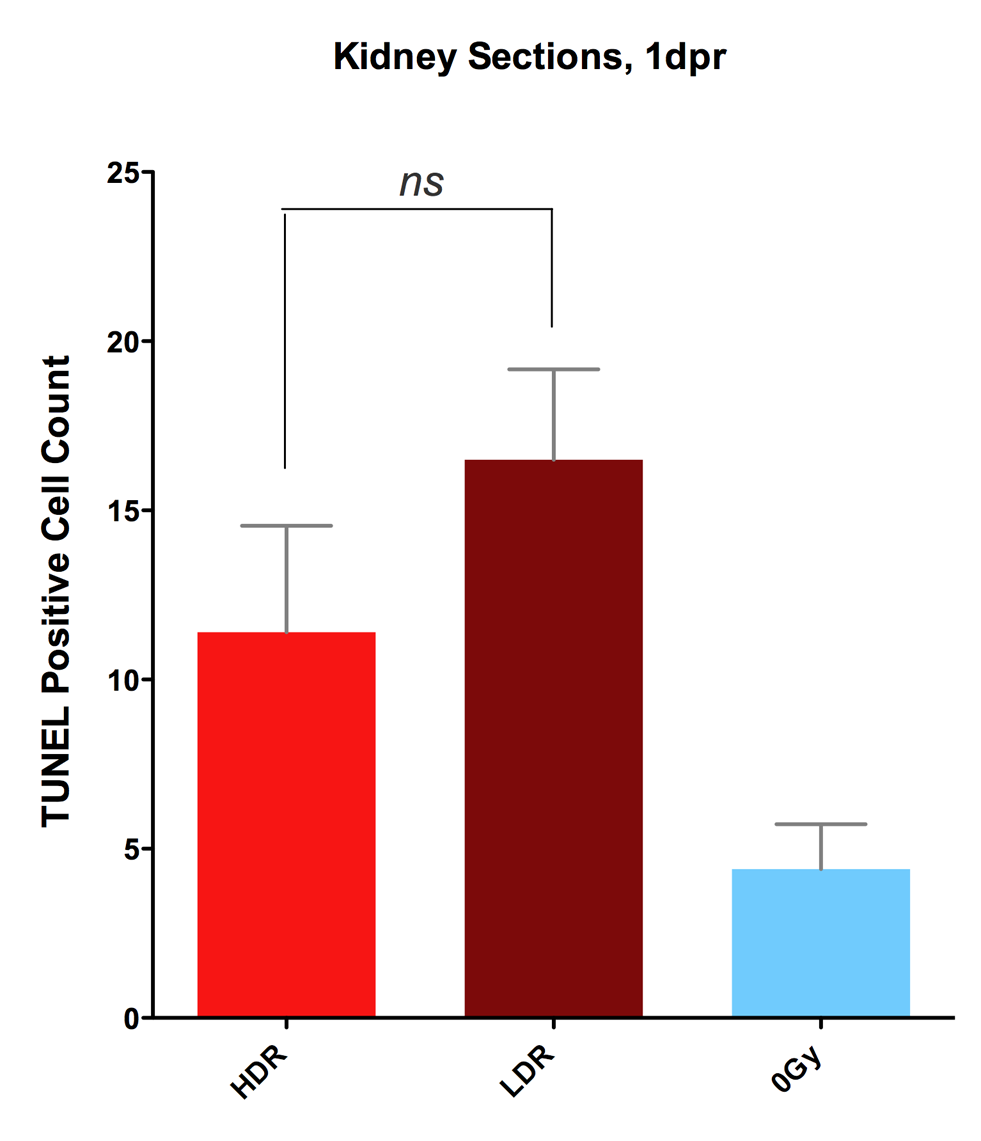

Supplement: Figure S1 — Quantification of TUNEL staining in Kidney sections. Photographs of head, saddle, and tail kidney regions were taken using a 40X objective. The numbers of TUNEL-positive cells were manually counted in each photograph. 3 fish and ten photographs were analyzed per group. Bars show mean and SEM. No significant difference was noted (unpaired t-test). (TIF) [file pone.0073745.s001.tif]

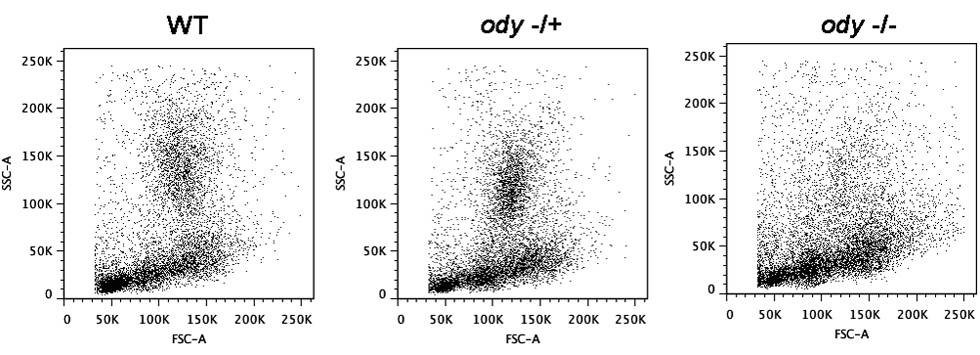

Supplement: Figure S2 — Hematopoietic Profiles of WT, ody −/+, and ody −/− Fish. Facs plots of hematopoietic cells pooled from kidneys of several fish of each genotype. Although heterozygous odysseus fish and WT fish both show lymphocyte, precursor, and myelomonocyte populations, myelomonocytes are conspicuously reduced in kidneys of homozygous odysseus mutants. (TIF) [file pone.0073745.s002.tif]
