# Supplementary material for: Effect of Radiation Dose-Rate on Hematopoietic Cell Engraftment in Adult Zebrafish
Source: PLoS One. 2013 Sep 18;8(9):e73745. doi: 10.1371/journal.pone.0073745 (PMC3776794; doi:10.1371/journal.pone.0073745)
Supplement: Supporting Text S1 — Primers. (DOCX) [file pone.0073745.s003.docx]

**qRT-PCR:** Taqman Primers used for this study were: *bactin1* (Dr03432610_m1), *ddb2* (Dr03429615_m1), *p53* (Dr03112089_m1), *cmyb* (Dr03432766_m1), *gata1a* (Dr03086722_m1), *mpx* (Dr03075669_m1), and *cxcl12a* (*sdf-1a)* (Dr03119119_m1).
